# Polysaccharide oxidation by lytic polysaccharide monooxygenase is enhanced by engineered cellobiose dehydrogenase

**DOI:** 10.1111/febs.15067

**Published:** 2019-10-01

**Authors:** Daniel Kracher, Zarah Forsberg, Bastien Bissaro, Sonja Gangl, Marita Preims, Christoph Sygmund, Vincent G. H. Eijsink, Roland Ludwig

**Affiliations:** ^1^ Department of Food Science and Technology BOKU – University of Natural Resources and Life Sciences Vienna Austria; ^2^ Manchester Institute of Biotechnology The University of Manchester Manchester UK; ^3^ Faculty of Chemistry, Biotechnology and Food Science Norwegian University of Life Sciences (NMBU) Ås Norway

**Keywords:** cellobiose dehydrogenase, cellulose degradation, copper monooxygenase, hydrogen peroxide, lytic polysaccharide monooxygenase

## Abstract

The catalytic function of lytic polysaccharide monooxygenases (LPMOs) to cleave and decrystallize recalcitrant polysaccharides put these enzymes in the spotlight of fundamental and applied research. Here we demonstrate that the demand of LPMO for an electron donor and an oxygen species as cosubstrate can be fulfilled by a single auxiliary enzyme: an engineered fungal cellobiose dehydrogenase (CDH) with increased oxidase activity. The engineered CDH was about 30 times more efficient in driving the LPMO reaction due to its 27 time increased production of H_2_O_2_ acting as a cosubstrate for LPMO. Transient kinetic measurements confirmed that intra‐ and intermolecular electron transfer rates of the engineered CDH were similar to the wild‐type CDH, meaning that the mutations had not compromised CDH’s role as an electron donor. These results support the notion of H_2_O_2_‐driven LPMO activity and shed new light on the role of CDH in activating LPMOs. Importantly, the results also demonstrate that the use of the engineered CDH results in fast and steady LPMO reactions with CDH‐generated H_2_O_2_ as a cosubstrate, which may provide new opportunities to employ LPMOs in biomass hydrolysis to generate fuels and chemicals.

## Graphical abstract

Lytic polysaccharide monooxygenases (LPMOs) require an electron donor and an oxygen species as a cosubstrate. We show that a single auxiliary enzyme, an engineered cellobiose dehydrogenase (CDH_oxy+_), provides both. CDH_oxy+_ produces about 30 times more H_2_O_2_ than the wild‐type CDH and greatly boosts the activity of LPMO. These results demonstrate H_2_O_2_‐driven LPMO activity and provide new opportunities for the application of CDH in industrial biomass utilization.

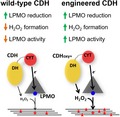

**Abbreviations**
AscA: ascorbic acid
CDH: cellobiose dehydrogenase
CYT: cytochrome domain of CDH
DH: dehydrogenase domain of CDH
FAD: flavin adenine dinucleotide
LPMO: lytic polysaccharide monooxygenase

## Introduction

Lytic polysaccharide monooxygenases (LPMOs, EC: http://www.chem.qmul.ac.uk/iubmb/enzyme/1/14/99/54.html53‐56, CAZy IDs: AA9‐11, AA13‐16) are copper‐containing enzymes found in chitin‐, starch‐, and plant cell wall‐degrading organisms 1, 2, 3, 4, 5. LPMOs use an unprecedented oxidative reaction mechanism to cleave and locally decrystallize these biopolymers, which enhances the activity of associated hydrolases 1, 2, 6, 7, 8. The reaction cycle starts with the reduction of LPMO’s mononuclear type‐II copper by external electron donors. After binding of a cosubstrate (O_2_ or H_2_O_2_), an oxygen atom is inserted into the C–H bond at the C1 or C4 carbon of a glycosidic bond in the substrate 9, 10, 11, which is broken by a spontaneous elimination reaction. The detailed catalytic mechanism of LPMOs and the nature of their cosubstrate are currently debated. Both O_2_ 12, 13, 14 and H_2_O_2_ 15, 16 are reported to interact with reduced LPMO, but with different implications on the reaction pathway. The monooxygenase reaction using O_2_ requires two sequential electron transfer steps and is likely to proceed via an intermittent dioxo 9, 12 or oxyl 17 species. In contrast, a single reduction step to yield Cu(I) could be followed by binding of H_2_O_2_ to directly form an oxyl‐ or a hydroxyl species 15. The reported turnover numbers of LPMO with H_2_O_2_ as cosubstrate are one to two orders of magnitude higher than those reported with O_2_ depending on the substrate and reaction conditions 15, 16, 18. However, exceeding H_2_O_2_ concentrations also lead to enzyme inactivation via autooxidation of, primarily, the Cu‐coordinating histidine residues 15. This makes a controlled supply of H_2_O_2_ essential to maintain LPMO stability during catalysis 16, 19.

In lignocellulose‐degrading fungi, one known route for LPMO reduction involves the electron transfer protein cellobiose dehydrogenase (CDH; EC: http://www.chem.qmul.ac.uk/iubmb/enzyme/1/1/99/18.html; CAZy ID: AA3_1) 6, 11, 20. CDHs are widely distributed extracellular flavocytochromes comprising a flavin adenine dinucleotide (FAD)‐containing dehydrogenase domain (DH) for substrate oxidation that transfers electrons to a mobile cytochrome *b* domain that can reduce the LPMO active site 9, 11, 20, 21. CDHs have a reported low oxidase activity 22, 23, 24, 25, for example, the *Crassicarpon hotsonii* (syn. *Myriococcum thermophilum*) CDH (*Ch*CDH) used in this study has a ca. 200 times lower turnover number for O_2_ than for flavin‐dependent 1,4‐benzoquinone reduction 26. Still, under physiological conditions, CDH might provide catalytically relevant amounts of H_2_O_2_ for LPMOs, which have a µm affinity for this cosubstrate 16, 27. To scrutinize the significance of H_2_O_2_ in CDH‐driven LPMO activity, we enhanced the H_2_O_2_ production rate of *Ch*CDH by means of genetic engineering. Our results demonstrate that a single synthetic enzyme, hereafter termed CDH_oxy+_, is able to fulfill a dual function as LPMO reductase and as a supplier of H_2_O_2_ for the LPMO reaction. This allowed reducing the effective concentration of CDH during oxidative polysaccharide degradation by a factor of ~ 30 relative to the wild‐type *Ch*CDH, while achieving similar LPMO activity. The results shed light on the role of H_2_O_2_ in LPMO catalysis and the CDH‐LPMO interplay.

## Results and Discussion

### Engineering of *Ch*CDH for increased oxygen reactivity

**Figure 1:**
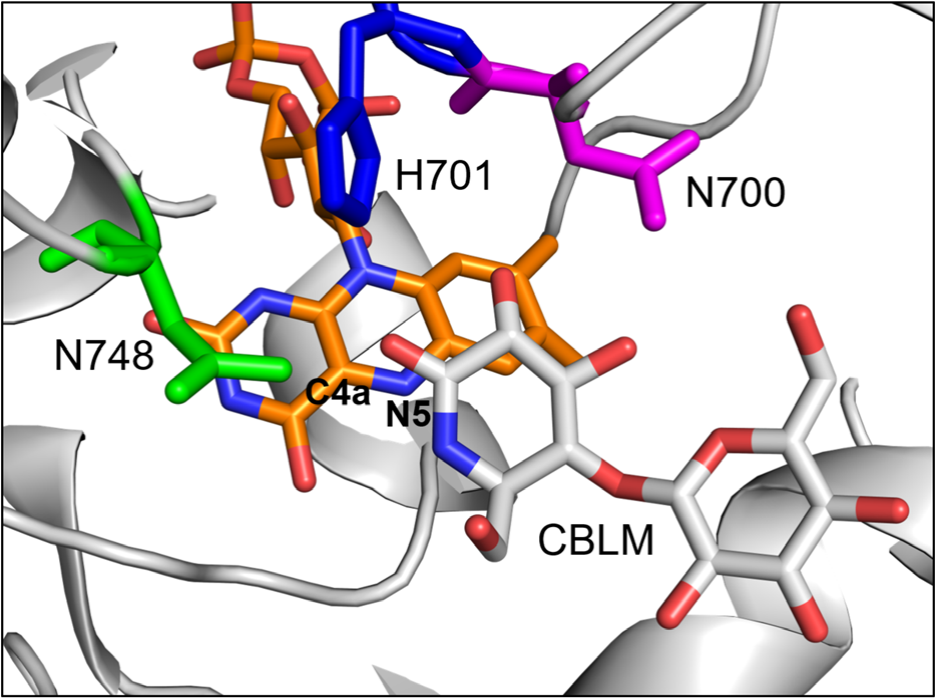
The active site of *Ch*CDH in complex with the inhibitor cellobionolactam (CBLM, pdb: http://www.rcsb.org/pdb/search/structidSearch.do?structureId=4QI5). Shown is the FAD‐cofactor (orange) with the indicated C4a and N5 atoms and the catalytic base H701 (blue). The positions N700 (magenta) and N748 (green) of the wild‐type *Ch*CDH were replaced in this study by serine and glycine, respectively.

We used a previously published variant of *C. hotsonii* CDH with a moderately (three times) increased H_2_O_2_ production rate (N700S) 28 as a template for semirational engineering. Further site‐saturation mutagenesis was performed on residue N748 since studies on other FAD‐dependent oxidases and monooxygenases had shown that this position on top of the isoalloxazine C4a position, which is adjacent to the reactive N5, influences the oxygen reactivity of flavoenzymes 29. The proposed reaction mechanism for the reduction of O_2_ proceeds via an initial electron transfer from the flavin hydroquinone to O_2_ forming a superoxide anion ($O_{2}^{-\cdot }$) followed by a spin inversion of the resulting caged radical pair. After spin inversion H_2_O_2_ is formed after a second electron transfer from the flavin semiquinone to the oxygen intermediate, which can proceed via the formation of either a transient C4a‐hydroperoxy‐flavin and subsequent decay to H_2_O_2_ or an outer sphere electron transfer 30, 31. The closest amino acid in CDH to the isoalloxazine C4a position is N748, which is also a neighbor of the catalytic H701 and therefore a hotspot for mutagenesis by influencing either the access of O_2_ to the FAD or the electron transfer steps (Fig. 1). A phylogenetic analysis of 56 *cdh* sequences showed that N748 is highly conserved in CDHs and only one sequence from a hitherto uncharacterized CDH contained a serine at the same position 32.

A site‐saturation library of N748 was transformed in *Pichia pastoris* X‐33 cells and was subjected to a high‐throughput screening using the peroxidase‐coupled ABTS (2,2'‐azino‐bis(3‐ethylbenzothiazoline‐6‐sulphonic acid)) assay for detection of H_2_O_2_ production 28. A second screening was based on the activity of CDH toward the artificial electron acceptor 2,6‐dichloroindophenol to normalize the expression level of the enzyme variants 28.

In active *Ch*CDH variants, the substitution N748G showed the highest activity with O_2_. The resulting double variant N748G/N700S was produced in both its full‐length form (CDH_oxy+_) and in a truncated form (DH_oxy+_), lacking the electron transferring cytochrome *b* domain 21 (Table 1). Steady‐state kinetic experiments showed a 27‐ and 36‐time higher specific activity of CDH_oxy+_ and DH_oxy+_, respectively, over the wild‐type enzymes (*Ch*CDH and *Ch*DH) when using the natural substrate cellobiose and O_2_ as electron acceptor (Table 1). The turnover of the non‐natural substrate lactose, which was used in later parts of this study for analytical reasons, was similarly enhanced. Interestingly, DH_oxy+_ showed a higher turnover than CDH_oxy+_, which indicates that the cytochrome domain can kinetically impair the two‐electron reduction of O_2_ by removing one electron from FADH_2_.

**Table 1:** Properties and catalytic activities of wild‐type and recombinant enzymes.

Enzyme/Variant	CYT domain	DH domain	Length (aa)	Mutations	Spec. activity. 2,6‐dichloroindophenol (U·mg^−1^)	Spec. act. O_2_ (U·mg^−1^)	
						Cellobiose	Lactose
*Ch*CDH	yes	yes	806	none	0.82	0.015 ± 0.001	0.017 ± 0.001
CDH_oxy+_	yes	yes	806	N700S/N748G	0.75	0.401 ± 0.019	0.368 ± 0.055
*Ch*DH	no	yes	576	none	1.42	0.016 ± 0.002	0.019 ± 0.002
DH_oxy+_	no	yes	576	N700S/N748G	1.37	0.580 ± 0.019	0.396 ± 0.043

### Activation of bacterial and fungal LPMOs by *Ch*CDH

**Figure 2:**
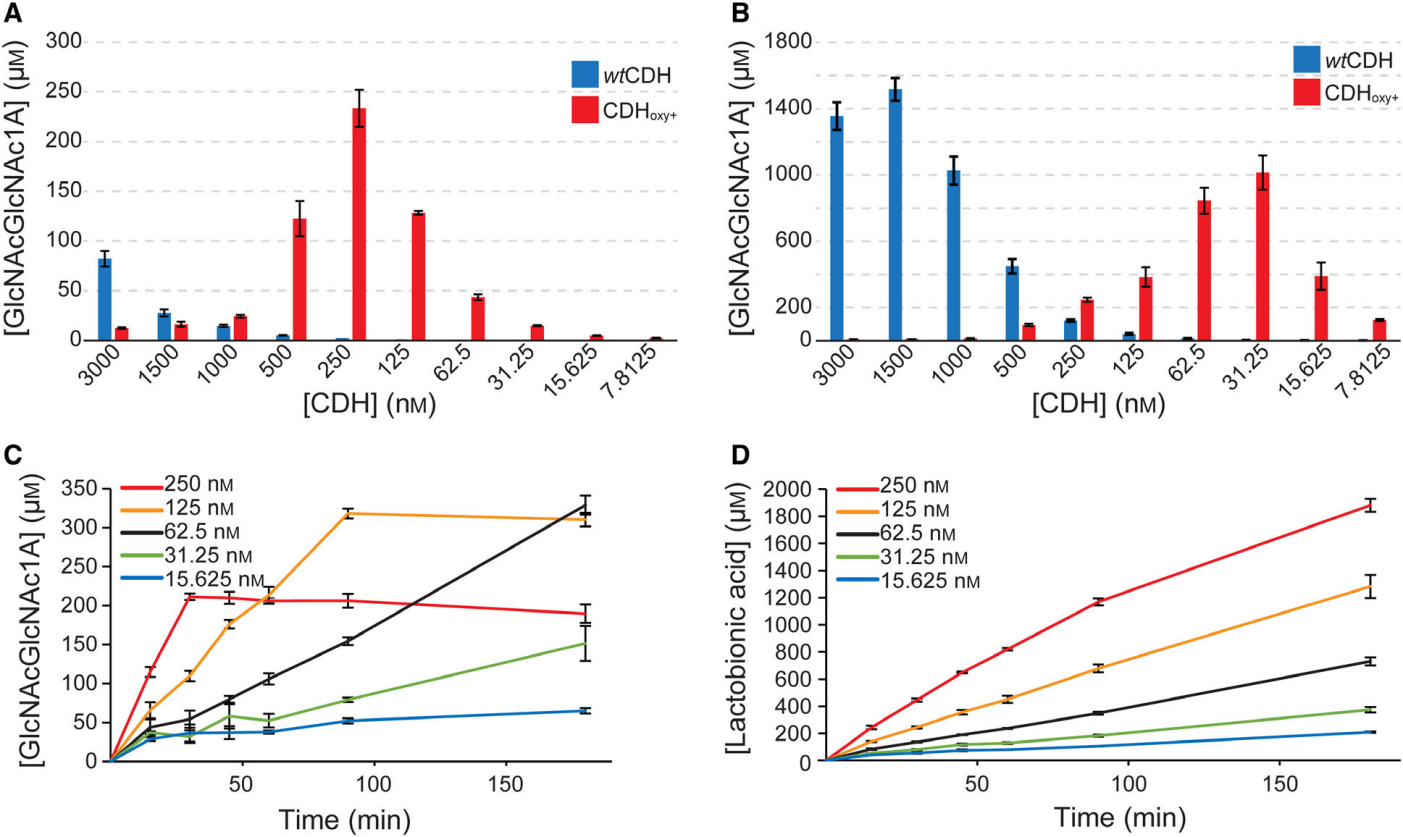
Product formation by *Sm*LPMO10A. Chitobionic acid concentration after incubation of β‐chitin with 1 µm *Sm*LPMO10A, 15 mm lactose and 10 different concentrations of *Ch*CDH (blue) or CDH_oxy+_ (red) after 1 h (A) or 24 h (B). Panel (C) shows the time‐dependent formation of chitobionic acid by *Sm*LPMO10A and (D) lactobionic acid by CDH_oxy+_ in reactions containing 1 µm LPMO and five different concentrations of CDH_oxy+_. All error bars show ± S.D. (*n* = 3).

We initially tested the ability of CDH_oxy+_ to activate the bacterial C1‐oxidizing chitin‐active LPMO10A from *Serratia marcescens* (Fig. 2), which releases C1‐oxidized chito‐oligosaccharides as a product of the chitin oxidation reaction. Using lactose as a CDH substrate allowed a clear distinction between oxidation products generated by the LPMO (chito‐oligosaccharides) and those generated by CDH (lactobionic acid) since the latter does not oxidize chito‐oligosaccharides 33.

CDH_oxy+_ was more effective in driving the LPMO reaction when compared to the wild‐type *Ch*CDH. For example, after 1 h, we observed a 2.8‐time higher LPMO product formation at a 12‐time lower CDH_oxy+_ concentration (Fig. 2A; compare bars for 3000 nm *Ch*CDH vs. 250 nm CDH_oxy+_), which translates into CDH_oxy+_ being ~ 35 times more efficient in driving the LPMO reaction. Comparison with product concentrations obtained after 24 h showed a ~ 30 times higher efficiency (Fig. 2B; 1500 nm *Ch*CDH vs. 31.25 nm CDH_oxy+_) but suggests considerable enzyme inactivation in reactions with higher CDH_oxy+_ concentrations. The nonlinear increase in the amount of reaction products from the 1‐ to 24‐h experiments and the lower optimal CDH : LPMO ratios found in the 24‐h experiment point toward a fast inactivation of one or both biocatalysts. We, therefore, analyzed the time‐dependent formation of oxidation products from both CDH (lactobionic acid) and LPMO (oxidized chito‐oligosaccharides). Data presented in Fig. 2C show that a high concentration of CDH_oxy+_ caused fast inactivation of the LPMO, while CDH activity was less affected (Fig. 2D). At low CDH_oxy+_ : LPMO ratios the LPMO activity was maintained throughout the reaction. This suggests LPMO inactivation when the rate of H_2_O_2_ production exceeded LPMO’s capacity to convert its reactive cosubstrate in a productive manner, leading to H_2_O_2_‐induced auto‐oxidation reactions 15.

**Figure 3:**
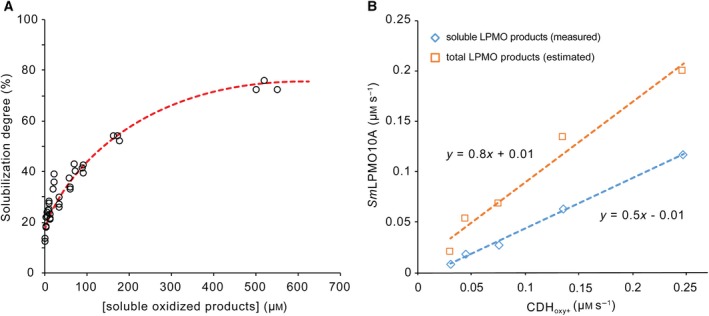
Oxidized sites generated during chitin degradation by *Sm*LPMO10A. Panel (A) shows a time‐independent plot of the degree of solubilization of oxidized products (i.e., the percentage of products obtained in the soluble fraction compared to the total number of oxidized sites) as a function of the quantity of detected solubilized oxidized products. The total number of oxidized sites was determined in reactions containing 10 g·L^−1^ β‐chitin and 1 µm *Sm*LPMO10A with varying concentrations of CDH_oxy+_ (31.25 or 125 nm) and 15 mm lactose. Samples were taken at different stages of the reaction (15–180 min). The samples were diluted twice and heat‐inactivated (100 °C, 15 min). Half of the reaction was filtrated and 1 µm *Sm*GH20 chitobiase was added for quantitation of solubilized products (i.e., the standard procedure for quantitation of solubilized oxidized sites). To the other half of the reaction, a cocktail of chitinases (containing 2 µm *Sm*Chi18A, 2 µm *Sm*Chi18C and 1 µm *Sm*GH20) was added followed by incubation for 20 h at 37 °C until the chitin was fully degraded. The resulting chitobionic acid concentration represents the total amount of oxidized sites. Rates derived from the initial phases of the progress curves in Fig. 2C,D only included time points where the solubilization was < 200 µm which means that the total number of oxidized sites is underestimated by ~ 50%. Panel (B) shows the LPMO rate without and with correction for this underestimation. The corrected curve shows that 1 molecule of lactose converted by CDH_oxy+_ leads to approximately 0.8 molecules of chitobionic acid being produced by *Sm*LPMO10A.

In all conversion experiments, the initial LPMO activity was linearly dependent on the H_2_O_2_ formation rate by CDH (Fig. 3). Quantitative analyses of both soluble and insoluble oxidized sites indicated that LPMO catalyzed approximately 0.8 reactions per CDH‐oxidized lactose molecule. The substoichiometric number in this experiment reflects CDH’s dual role as a source of the cosubstrate and as an electron donor for LPMO.

**Figure 4:**
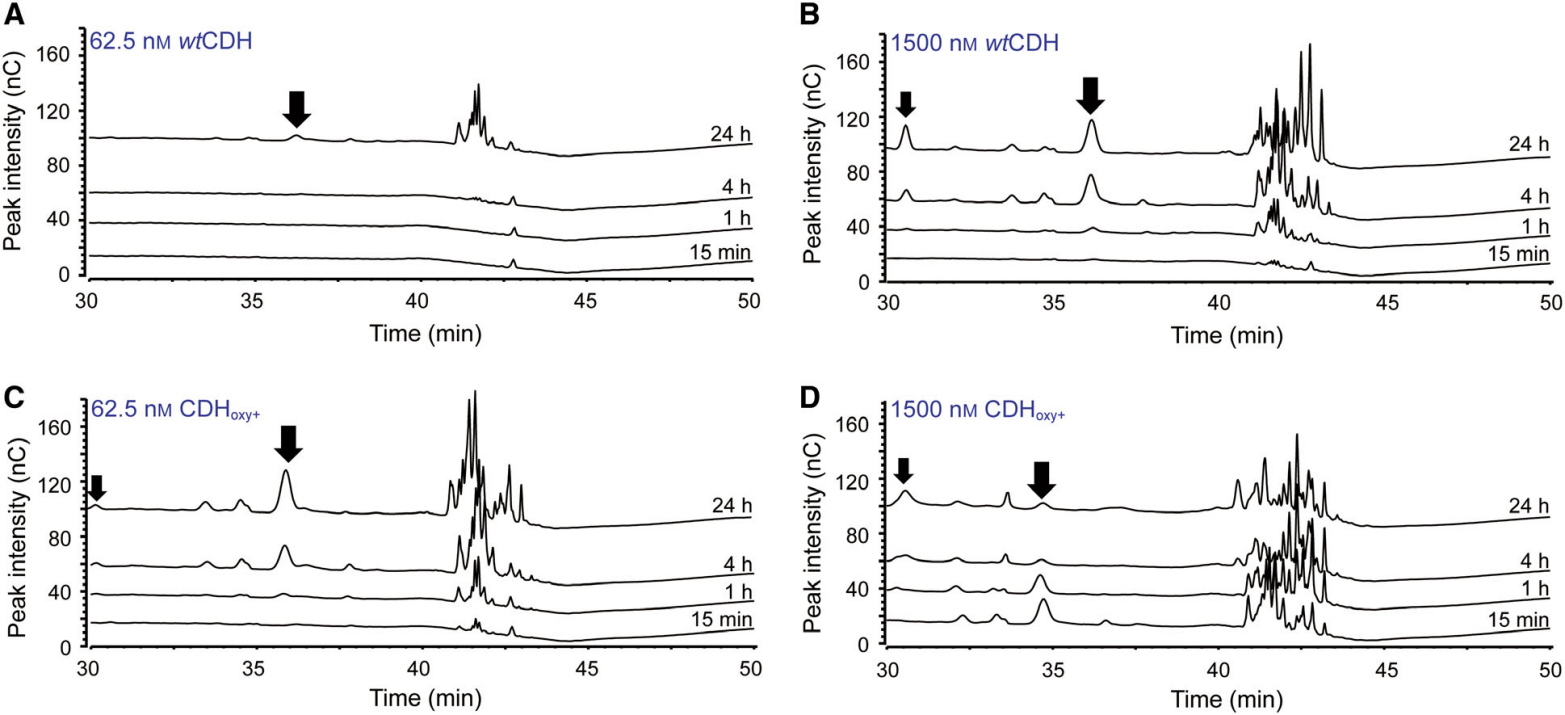
Product formation by *Nc*LPMO9C. HPAEC‐PAD chromatograms show C4‐oxidized products generated in reactions containing 1 µm *Nc*LPMO9C in the presence of 62.5 nm (A) or 1500 nm *Ch*CDH (B) and 62.5 nm (C) or 1500 nm CDH_oxy+_ (D). All reactions contained 5 g·L^−1^ PASC and 15 mm lactose and were incubated in 50 mm potassium phosphate buffer, pH 6.0, for up to 24 h in an Eppendorf thermomixer (Hamburg, Germany) set to 30 °C and 800 r.p.m. Samples were heat‐inactivated (15 min, 100 °C) and filtered (0.22 µm) prior to analysis. The bigger black arrows indicate the Glc4GemGlc C4‐oxidized product, whereas the smaller black arrows, seen in panel (B–D), show Glc_2_Glc1A peaks that are formed as a result of CDH oxidation of the native cellotriose that is formed by the LPMO upon cleavage of shorter cello‐oligosaccharides. Peak annotations are based on earlier studies 41, 42. Note that the elution times vary slightly between different runs. Double (C1/C4) oxidized products (peaks eluting after ~ 40 min) dominate since CDH oxidizes the reducing end of the C4‐oxidized products formed by the LPMO. Note that C4‐oxidized products are unstable, which explains why some peaks become smaller over time in panel (D).

When using the fungal *Nc*LPMO9C in combination with *Ch*CDH or CDH_oxy+_, product chromatograms were dominated by double (C1/C4) oxidized products (Fig. 4) since CDH oxidizes the reducing end of the C4‐oxidized products formed by the LPMO. The complex product spectrum of this reaction and the lack of suitable standards did not allow for a thorough analysis of the reaction kinetics. However, when comparing overall product peaks it becomes obvious that compared to reactions with *Ch*CDH an ~ 24 time lower concentration of CDH_oxy+_ generated a similar product concentration (compare Fig. 4B vs. C). Note that in the reaction with high concentrations of CDH_oxy+_ (1.5 µm; Fig. 4B) *Nc*LPMO9C became rapidly inactivated, which corroborates the findings with *Sm*LPMO10A.

### Electron transfer rates of *Ch*CDH and CDH_oxy+_

**Figure 5:**
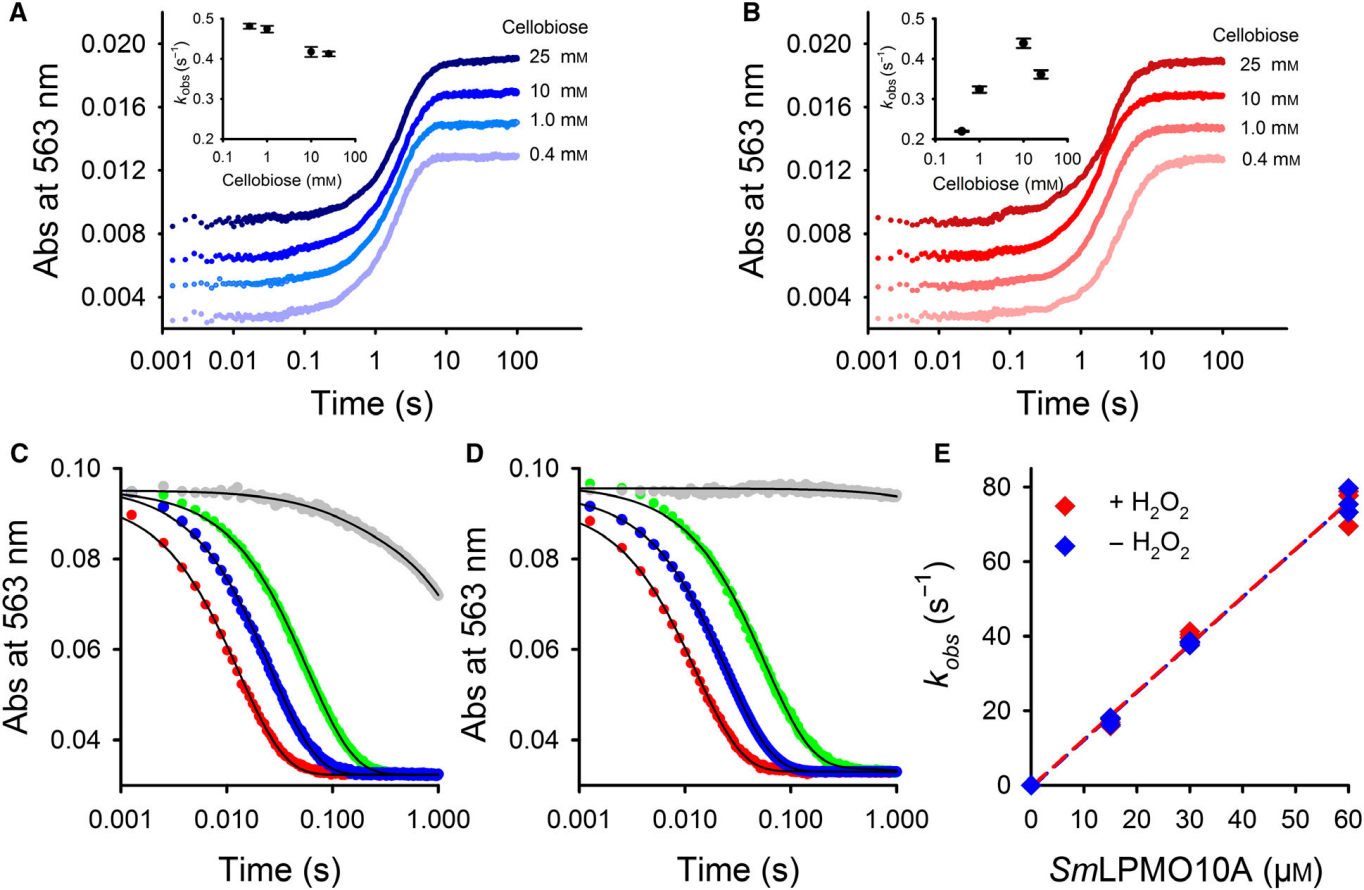
Electron transfer between redox centers. Intramolecular electron transfer in *Ch*CDH (A) and CDH_oxy+_ (B) from FAD, in the DH domain, to heme *b*, in the cytochrome domain, at the indicated cellobiose concentrations. Heme *b* reduction was followed at 563 nm (α‐band). The concentration of CDH after mixing was 1.5 µm. Prior to all measurements, substrate and enzyme solutions were carefully degassed by applying alternating cycles of vacuum and nitrogen pressure to avoid interference with atmospheric oxygen. All measurements were carried out at 30 °C in 50 mm sodium phosphate buffer, pH 6.0. The insets show the cellobiose concentration‐dependent electron transfer rates. Panels (C) and (D) show raw traces for heme *b* reoxidation by *Sm*LPMO10A in the absence (catalase added, C) or presence (no catalase added, D) of H_2_O_2_ using 0 µm (gray), 15 µm (green), 30 µm (blue) or 60 µm (red) LPMO. (E) Electron transfer from reduced heme *b* to LPMO in the presence (red) or absence (blue) of approximately 250 µm H_2_O_2_ formed by CDH during prereduction. All traces are the average of triplicate measurements. Error bars show ± S.D (*n* = 3).

We conducted rapid mixing experiments to probe CDH’s internal electron transfer from the reduced FADH_2_ to heme *b*. To this end, we mixed *Ch*CDH and CDH_oxy+_ with an excess of cellobiose and monitored both the reduction of the FAD (449 nm) and the heme *b* (563 nm) cofactors. For both CDH_oxy+_ and *Ch*CDH the intramolecular electron transfer rates were similar at cellobiose concentrations higher than 1 mm (0.35–0.48 s^−1^) (Fig. 5A). The lower electron transfer rates for CDH_oxy+_ at low cellobiose concentrations indicate competition for electrons between O_2_ and the cytochrome domain at the DH domain (Fig. 5B).

The second, intermolecular electron transfer step from the reduced heme *b* to LPMO was probed by reacting prereduced CDH_oxy+_ with oxidized LPMO (Fig. 5C–E). Prereduction of CDH with cellobiose was performed under atmospheric conditions with or without catalase to investigate the effect of the by‐product H_2_O_2_ on the observed rate. The presence of H_2_O_2_ had no effect on the LPMO reduction rate and in both cases, the apparent bimolecular rate constant for the CDH_oxy+_/LPMO interaction was 1.27 × 10^6^ m ^−1^·s^−1^, which is similar to the reported value for *Ch*CDH/LPMO (0.63 × 10^6^ m ^−1^·s^−1^; 33).

These experiments show that both *Ch*CDH and CDH_oxy+_ have similar electron transferring capabilities under the tested conditions. We, therefore, conclude that the increased ability of CDH_oxy+_ to support LPMO reactions is not caused by changes in the ability to reduce the LPMO but to increase production of H_2_O_2_.

### Activation of LPMO by *Ch*DH and DH_oxy+_

**Figure 6:**
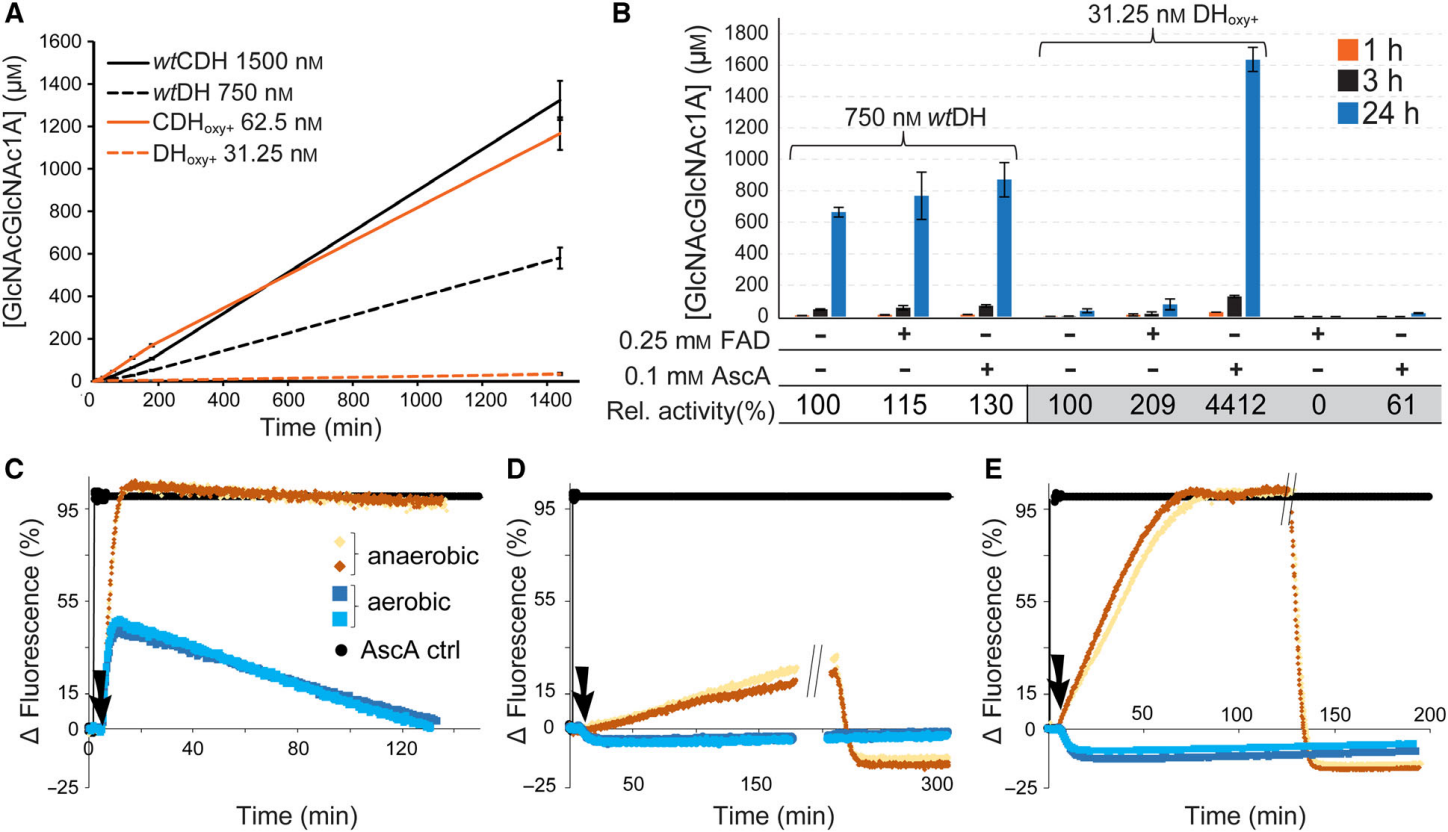
LPMO activity driven by CDH without the cytochrome domain. (A) Time courses of chitobionic acid production by 1 µm *Sm*LPMO10A in combination with CDH variants. (B) Quantitation of LPMO products generated in reactions containing 0.25 mm FAD or 0.1 mm ascorbic acid (AscA) in the presence or absence of 750 nm *Ch*DH or 31.25 nm DH_oxy+_. Relative activities show the enhancement upon addition of FAD or AscA (based on product levels after 24 h). The error bars show ± S.D (*n* = 3). Lower panels: Reduction of *Sm*LPMO10A by different CDH variants under anaerobic or aerobic conditions. All reactions contained 2 µm *Sm*LPMO10A and 0.25 µm *Ch*CDH (C); 0.25 µm *Ch*DH (D); or 31.25 nm DH_oxy+_ (E). Reactions were initiated by addition of lactose (1 mm final concentration, black arrow) and were performed in duplicate (both traces are displayed). The variation in fluorescence is relative to the maximum increase in fluorescence measured for a control reaction (black line) in which lactose was replaced by 10 µm AscA, which represents a 100% reduction of *Sm*LPMO10A. The " //" labels in panel (D) and (E) indicate when the reactions were no longer under anaerobic conditions.

**Figure 7:**
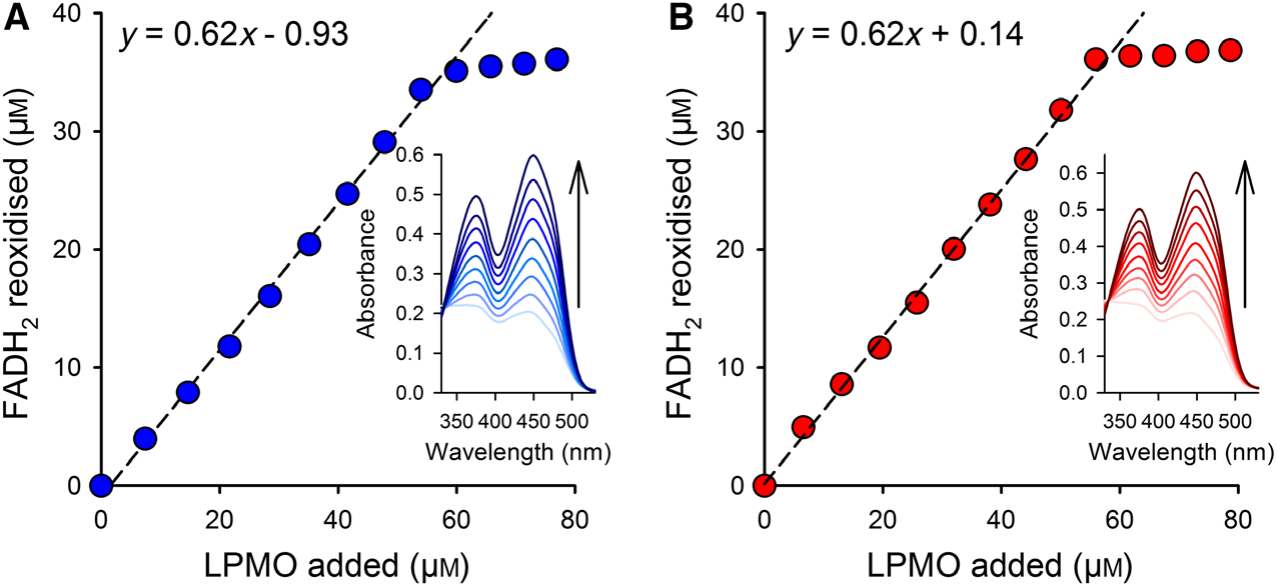
Stoichiometry of LPMO reduction with FADH_2_. Titration of FADH_2_ with *Nc*LPMO9C (A) or *Sm*LPMO10A (B). Oxidized FAD (60 µm) was approximately 70% reduced with sodium dithionite. The FADH_2_ was reoxidized with LPMO by adding aliquots of 3 µL (*Nc*LPMO9C; 515 µm) or 1.5 µL (*Sm*LPMO10A; 906 µm) to the cuvette. Absorption spectra were recorded with an Agilent 8453 UV/VIS‐Spectrometer featuring a diode array detector. Blank reactions (buffer titrated to FADH_2_ or LPMO) were subtracted. The concentration of FAD and LPMOs was determined based on their molar absorption coefficients (FAD ε_450_ = 11.3 mm ^−1^·cm^−1^; *Nc*LPMO9C ε_280_ = 46.91 mm ^−1^·cm^−1^; *Sm*LPMO10A ε_280_ = 35.20 mm ^−1^·cm^−1^). All reactions were carried out at 23 °C in an anaerobic glove box (Whitley DG250, Don Whitley Scientific) flushed with a nitrogen/hydrogen mixture. The data show that, for both LPMOs, about two molecules of enzyme were required to reoxidize 1 molecule of FADH_2_.

To gain further insight into the CDH‐LPMO interplay, we replaced the full‐length enzymes in conversion experiments with DH_oxy+_ and *Ch*DH at concentrations (31.25 and 750 nm, respectively) expected to produce the same amount of H_2_O_2_. Unexpectedly, in the absence of an additional reductant like ascorbic acid (AscA), both dehydrogenase domains were capable of driving the LPMO reaction to some extent, despite the lack of an electron transferring cytochrome domain (Fig. 6). The DH domain of *Ch*CDH was previously shown to be incapable of direct electron transfer to LPMO 21, but activation of LPMO by some flavoenzymes without cytochrome domains under steady‐state conditions has been reported 34. Considering that all the investigated flavoenzymes bind FAD noncovalently and that in our experiments *Ch*DH (at 750 nm) was much more effective than DH_oxy+_ (at 31.25 nm) at initiating LPMO activity (Fig. 6A), we considered FAD itself as a potential redox mediator between LPMO and DH. Thus, as a control, we carried out reactions with added FAD and compared it with reactions supplemented with the commonly used reductant AscA. Adding 0.25 mm FAD or 0.1 mm AscA to reactions containing 750 nm *Ch*DH led to a slight increase in the amount of formed LPMO reaction products (Fig. 6B). A doubling of LPMO activity was observed upon adding FAD to reactions containing 31.25 nm DH_oxy+_, whereas in this case, the addition of 0.1 mm AscA led to a much higher, 55‐fold, increase in the LPMO product concentration after 24 h. These observations show that FAD can act as a redox mediator between the DH domain and the LPMO. The strong effect of AscA on the reactions with DH_oxy+_ demonstrates that at low DH concentrations the concentration of dissociated FAD, and, thus, reduction of the LPMO becomes rate limiting. In this case, the addition of another reductant (AscA) alleviates the limitation and enables the LPMO to convert the available H_2_O_2_. Titrations of FADH_2_ with oxidized *Sm*LPMO10A and *Nc*LPMO9C (Fig. 7) confirmed that FADH_2_ indeed reduces both enzymes with the expected 1 : 2 stoichiometry (1 electron per LPMO active site copper), underpinning the potential of FADH_2_/FAD redox cycling between DH and LPMO. Whether dissociated FADH_2_ is of relevance as a natural redox mediator system is debatable, since the DH concentration in the experiments was higher than those usually observed *in vivo*.

We also considered that superoxide, generated via a one‐electron reduction of O_2_ at the cytochrome domain, could act as a potential reductant for LPMO. Such a reduction was previously shown to occur at high superoxide concentrations, albeit with low efficiency 15. Using a fluorescence‐based assay 35 we observed that under anaerobic conditions *Ch*CDH reduced LPMO completely, while under aerobic conditions only 45% of the LPMO was reduced due to simultaneous reoxidation of CDH by O_2_ (Fig. 6C). The fact that both *Ch*DH and DH_oxy+_ reduced LPMO under anaerobic conditions (Fig. 6D,E) indicates that LPMO reduction by these truncated enzymes does not involve superoxide and is rather due to FADH_2_/FAD redox cycling, as discussed above.

## Conclusions

Taken together, these experiments support the importance of H_2_O_2_‐driven LPMO catalysis. While *Ch*CDH and CDH_oxy+_ transfer electrons to the LPMO with similar efficiencies, they produce different amounts of H_2_O_2_, which translates into different LPMO activities. In accordance with the correlation between H_2_O_2_ production by the CDH and LPMO activity, it has been noted earlier that the rate of *Ch*CDH‐driven LPMO activity is similar to the rate of H_2_O_2_ production by *Ch*CDH in reactions with O_2_ as the only electron acceptor 33. It has been suggested that H_2_O_2_‐driven LPMO activity implies that, when once reduced, an LPMO could carry out multiple oxidative cleavages 15. The apparent stoichiometry of the reactions that may be derived from the data displayed in Fig. 3 is compatible with these previous observations: our data show that approximately 8 out of 10 oxidized substrate molecules of CDH_oxy+_ are used to generate H_2_O_2_, which is stoichiometrically used for polysaccharide cleavage. The remaining two substrate molecules can generate four reduction equivalents for the reduction of LPMO, meaning that at least two H_2_O_2_ molecules are consumed per LPMO reduction. Studies with externally added reductant and H_2_O_2_ have shown that under certain conditions a reduced LPMO may catalyze 15–18 reactions 15, 36.

The cytochrome domain allows for specific intermolecular electron transfer from CDH to LPMO, but LPMO reduction can also be accomplished by a small molecule reductant like AscA or, interestingly, also by FADH_2_ when the flavodehydrogenase is applied at high concentrations. The exact mechanism of LPMO activation is currently disputed 15, 18. In light of this debate, it is worth noting the AscA experiments depicted in Fig. 6B: the amount of oxidized products generated after 24 h in a reaction containing 0.1 mm AscA and 31.25 nm DH_oxy+_ was 72 times higher when compared to a reaction containing only 0.1 mm AscA. This difference supports the hypothesis that under commonly employed reaction conditions in LPMO experiments the production of H_2_O_2_ is rate limiting.

Aside from providing novel insight into H_2_O_2_‐driven LPMO catalysis and providing a newly engineered enzyme, CDH_oxy+_, with potential applications in industrial biomass processing, the present study sheds light on a rarely investigated biological role of CDH. The very low O_2_ turnover of CDHs might provide a slow but steady H_2_O_2_ supply for LPMOs in a physiological setting, thereby avoiding oxidative damage of LPMO.

## Materials and methods

### Generation of* Ch*CDH and CDH_oxy+_

The codon‐optimized expression plasmid pPIC‐MtSopt encoding the full‐length wild‐type CDH from the thermophilic fungus *C. hotsonii* (syn. *M. thermophilum*; *Ch*CDH) under the control of the methanol‐inducible AOX promoter was used as a parental plasmid for the generation of CDH_oxy+_. In a previous study, we already used this plasmid as a template for a semirational approach resulting in variant N700S with a moderately improved H_2_O_2_ production rate 28. In the present study, the plasmid pPIC‐MtCDH N700S encoding the above‐mentioned variant was used as the starting point for an additional round of protein engineering by site‐saturation mutagenesis. A *cdh* library containing all possible codons at the targeted position N748 was prepared using the forward primer: TACCACTNNSCCAACTTCTTACATTGTCG and the reverse primer: AAGTTGGSNNAGTGGTAGGAACACC. A library of 368 clones was screened by a differential screening using the peroxidase‐coupled ABTS assay for detection of H_2_O_2_ production and the 2,6‐dichloroindophenol assay to normalize the expression level of the enzyme variants 28. In this screening, five variants showed an increased H_2_O_2_ production compared to the parental enzyme N700S. The sequencing results showed that all of them carried the additional mutation N748G. With respect to its increased oxygen reactivity, the identified *Ch*CDH variant N700S/N748G was named CDH_oxy+_.

### Generation of* Ch*DH and DH_oxy+_

To obtain CDH and its oxygen‐converting variant without the electron‐transferring cytochrome domain, two truncated sequences (expression plasmids pPIC‐wtDH and pPIC‐DH_oxy+_) were synthesized by a commercial provider (ATG:biosynthetics, Merzhausen, Germany). In both plasmids, the sequence encoding for the native signal sequence (nucleotides 1–63) was directly followed by the dehydrogenase domain encoding sequence [nucleotides 750–2481 (F230—L806)]. The 687 nucleotides encoding for the cytochrome domain and the interdomain linker (N1—S229) were omitted in pPIC‐wtDH. The expression plasmid pPIC‐DH_oxy+_ additionally carried the two mutations N700S and N748G.

### Heterologous production and purification

All four enzymes were recombinantly produced in *P. pastoris* X‐33 cells and purified as previously reported 37. Table 1 shows the activities of the CDH and DH variants which were measured with 2,6‐dichloroindophenol as a chromogenic electron acceptor and 30 mm lactose as a substrate in 50 mm potassium phosphate buffer, pH 6.0. Data are expressed as mean values ± SD from three measurements. The calculated molar absorption coefficients at 280 nm given in Table 1 were used to calculate the protein concentrations and the specific activities.

The LPMO 10A from *S. marcescens* (*Sm*LPMO10A, also known as CBP21) was recombinantly produced in *Escherichia coli* and purified by chitin affinity chromatography as previously described 38. A threefold molar surplus of Cu(II)SO_4_ was added to the pure protein to ensure that each LPMO molecule has a copper cofactor. Excess copper was removed by applying the LPMO‐copper solution on a PD Midi‐Trap G‐25 column (GE Healthcare, Vienna, Austria) equilibrated with 50 mm potassium phosphate buffer, pH 6.0. The protein concentration was determined by measuring A280 using the calculated molar absorption coefficient of 35.20 mm ^−1^·cm^−1^.

The LPMO 9C from *Neurospora crassa* (*Nc*LPMO9C, previously termed PMO‐02916) was recombinantly produced in *P. pastoris* in a bioreactor and purified by a two‐step chromatographic procedure as previously described 39. The purified enzyme solution was repeatedly diafiltered against 50 mm potassium phosphate buffer, pH 6.0. The protein concentration was determined by measuring A280 using the calculated molar absorption coefficient of 46.91 mm ^−1^·cm^−1^.

### Oxygen reactivity of CDH variants

The oxygen turnover of *Ch*CDH and CDH variants was measured by incubating either 1.5 µm of the CDH and DH with low oxygen turnover (*Ch*CDH and *Ch*DH) or 30 nm of the CDH and DH variants with high oxygen turnover (CDH_oxy+_ and DH_oxy+_) with 5 mm cellobiose or lactose. Reactions had a total volume of 500 µL and were incubated at 40 °C in 50 mm sodium phosphate buffer, pH 6.0, at a constant agitation of 800 r.p.m. Samples were withdrawn regularly, incubated at 95 °C for 10 min to stop the reaction and measured by HPLC for aldonic acid formation.

### Standard LPMO/CDH reactions

Standard reactions were set up with 1 µm *Sm*LPMO10A and 10 g·L^−1^ β‐chitin, 15 mm lactose and the different CDH variants (i.e., *Ch*CDH, CDH_oxy+_, *Ch*DH, or DH_oxy+_) with concentrations varying from 7.8 to 3000 nm. When using the truncated DH variants, lower concentrations were used as these variants produce more H_2_O_2_ compared to the full‐length enzymes. All reactions were carried out in 50 mm potassium phosphate buffer, pH 6.0, in a Thermomixer set to 30 °C and 800 r.p.m. At regular intervals, samples were taken from the reactions, and the LPMO activity was stopped by directly separating the soluble fractions from the insoluble chitin particles by filtration using a 96‐well filter plate (Millipore, Burlington, MA, USA) operated with a vacuum manifold. For samples where both aldonic acids from the LPMO and CDH (i.e., chitobionic acid and lactobionic acid) were subjected to quantitative analysis, the enzyme reaction was first stopped by 15 min heat inactivation at 100 °C followed by filtration (0.22 µm). *Sm*LPMO10A‐generated chito‐oligosaccharides were further degraded by incubation with 1 µm *Sm*GH20 chitobiase as previously described 40 to produce *N*‐acetyl‐glucosamine and chitobionic acid, where the latter product represents the sum of all solubilized oxidized sites. To determine the total oxidized products, the LPMO reactions were stopped by heat inactivation (100 °C, 15 min) followed by 20 h of incubation with 2 µm of the *S. marcescens* exo‐chitinase A (*Sm*Chi18A) and 2 µm endo‐chitinase (*Sm*Chi18C) as well as 1 µm *Sm*GH20 chitobiase.

### Quantitation of aldonic acids

Aldonic acids generated by CDH were quantified following two previously published HPLC methods 41, 42. Quantitation of GlcNAcGlcNAc1A (i.e., chitobionic acid) from reactions containing *Sm*LPMO10A was accomplished using an RSLC system (Dionex, Sunnyvale, CA, USA) equipped with a 100 × 7.8‐mm Rezex RFQ‐Fast Acid H+ (8 %) (Phenomenex, Torrance, CA, USA) column operated at 85 °C. Samples of 8 µL were injected into the column and solutes were eluted isocratically using 5 mm sulfuric acid as mobile phase with a flow rate of 1 mL·min^−1^. The elution of products was monitored and recorded at 194 nm.

In‐house made chitobionic acid solutions with known concentrations were used to generate a standard curve of oxidized products. Briefly, (GlcNAc)_2_ was dissolved to a final concentration of 5.0 mm in 50 mm Tris/HCl pH 8.0 and incubated overnight with 0.12 mg·mL^−1^ *Fusarium graminearum* chito‐oligosaccharide oxidase 43 at 20 °C as previously described 40 to generate chitobionic acid (i.e., GlcNAcGlcNAc1A).

### Rapid kinetic studies of cellobiose dehydrogenase

Rapid kinetic experiments were performed with an SX20 stopped‐flow spectrophotometer (Applied Photophysics, Leatherhead, UK) using a photomultiplier tube (PMT) detector. All experiments were performed in 50 mm sodium phosphate buffer, pH 6.0, at 30 °C. Substrate and enzyme solutions were degassed prior to all measurements unless stated otherwise. The cellobiose‐dependent reduction of the CDH cytochrome domain was measured in single mixing mode by following the absorbance at 563 nm (heme α‐band). For the determination of the interdomain FAD‐to‐heme *b* electron transfer, CDH and cellobiose were used at final concentrations of 1.5 µm and 0.4–25 mm, respectively. Electron transfer from the reduced heme *b* to *Sm*LPMO10A was studied in sequential mixing mode. Initially, CDH (20 µm) was mixed with 5 mm of cellobiose in an aging loop until the full reduction of the heme *b* cofactor was observed (320 s). The reduced enzyme was rapidly mixed with *Sm*LPMO10A (15–60 µm) or buffer. These experiments were performed aerobically at a dissolved oxygen concentration of 250 µm. Approximately the same concentration of H_2_O_2_ was accumulated during prereduction of CDH with cellobiose in the aging loop. The same experiments were therefore performed in the presence of 500 U·mL^−1^ catalase from *Corynebacterium glutamicum* (Sigma Aldrich, St. Louis, MO, USA) in the aging loop to remove H_2_O_2_ before reacting the enzyme with LPMO. The required aging time in these experiments was 360 s. Observed rates (*k*_obs_) were determined by fitting the initial slope of at least three experimental repeats to an exponential function using the pro data sx software (Applied Photophysics).

### Titration of* Sm*LPMO10A and* Nc*LPMO9C with FADH_2_

All experiments were performed in 50 mm sodium phosphate buffer, pH 6.0, in an anaerobic glove box (Whitley DG250; Don Whitley Scientific, Shipley, UK), which was continuously flushed with a nitrogen/hydrogen mixture (90/10). Residual oxygen was removed with a palladium catalyst and the generated water vapor was absorbed by silica gel. To probe the interaction of FADH_2_ with LPMO, oxidized FAD (60 µm) was approximately 70% reduced with sodium dithionite to avoid an excess of reductant. FADH_2_ was reoxidized with LPMO by adding aliquots of 3 µL Cu(II)‐loaded *Nc*LPMO9C from a 515 µm stock solution or 1.5 µL Cu(II)‐loaded *Sm*LPMO10A from a 906 µm stock solution to the cuvette. Spectra were recorded after mixing. Blank runs containing FADH_2_ in buffer or LPMO in buffer were subtracted from these spectra. Electronic absorption spectra were recorded with an Agilent 8453 UV‐visible spectrophotometer (Santa Clara, CA, USA) equipped with a photodiode array detector. All reagents and enzyme solutions used in the glove box were extensively degassed by applying alternate cycles of vacuum and nitrogen pressure. All experiments were carried out in quartz cuvettes (200 µL total volume) at a constant temperature of 25 °C.

### Monitoring the redox state of *Sm*LPMO10A by fluorimetry

All reactions were carried out in 50 mm potassium phosphate buffer, pH 6.0 at 25 °C, prepared under aerobic or anaerobic conditions, and contained 2 µm *Sm*LPMO10A mixed with either *Ch*CDH (0.25 µm) or *Ch*DH (0.25 µm) or DH_oxy+_ (31.25 nm). Enzyme mixtures were transferred to a sealable fluorescence quartz cuvette equipped with screw cap and septum (Hellma, Müllheim, Germany), which was inserted into a Cary Eclipse Fluorescence spectrophotometer (Agilent Technologies, Santa Clara, CA, USA). Excitation and emission wavelengths were set to 280 and 340 nm, respectively, and the PMT detector voltage set to 550 V, as previously described 35. Reactions were initiated by addition of lactose (1 mm final concentration) using a Hamilton syringe. In a control reaction, carried out in anaerobic conditions, the lactose/CDH system was replaced by AscA (10 µm final concentration) to reduce the LPMO, providing a reference fluorescence signal corresponding to 100% reduction of *Sm*LPMO10A. All fluorescence signals were expressed as the variation in fluorescence relative to the maximum increase observed in this control reaction.

## Conflict of interest

The authors declare no conflict of interest.

## Author contributions

DK planned, performed, and interpreted oxygen conversion, rapid kinetic and anaerobic titration experiments, and wrote the original draft of the manuscript; ZF planned, performed, and interpreted conversion and electron transfer experiments and contributed to the original draft; BB planned, performed, and interpreted oxidation studies and fluorescence experiments; SG supported conversion and oxidation studies; MP and CS generated, produced, and purified the CDH variants and characterized them; VGHE and RL equally planned the study, acquired funding and administrated the project, supported the evaluation and interpretation of experiments, and wrote the final version of the manuscript.
